# Agreement Between Intraoperative Findings and Histopathological Diagnosis and Their Association with Postoperative Outcomes in Acute Appendicitis

**DOI:** 10.3390/diagnostics16101463

**Published:** 2026-05-11

**Authors:** Ivan Maleš, Anđela Šarić, Ivan Lovrinčević, Joško Božić, Zenon Pogorelić

**Affiliations:** 1 Department of Abdominal Surgery, University Hospital of Split, Spinčićeva 1, 21000 Split, Croatia; 2 Department of Surgery, School of Medicine, University of Split, Šoltanska 2A, 21000 Split, Croatia; 3 Department of Pediatric Surgery, University Hospital of Split, Spinčićeva 1, 21000 Split, Croatia; 4 Department of Pathophysiology, School of Medicine, University of Split, Šoltanska 2A, 21000 Split, Croatia

**Keywords:** acute appendicitis, appendectomy, histopathology, intraoperative findings, postoperative outcomes, length of hospital stay, antibiotic therapy, pediatric surgery, readmission, complications

## Abstract

**Background/Objectives:** Intraoperative assessment guides the immediate postoperative management of acute appendicitis, whereas histopathological confirmation becomes available only after key clinical decisions have been made. This study evaluated the agreement between intraoperative and histopathological grading of acute appendicitis and compared their associations with postoperative outcomes, particularly length of hospital stay (LOS) and duration of antibiotic therapy. **Methods:** This retrospective single-center study included pediatric and adult patients who underwent appendectomy for suspected acute appendicitis at the University Hospital of Split between 1 January 2020 and 31 December 2025. After exclusion criteria were applied, 2279 patients were included. Agreement between intraoperative and histopathological classifications was assessed using Cohen’s kappa and weighted kappa. Associations with postoperative outcomes were examined using Kendall’s tau correlation, bootstrap comparison between age groups, Kruskal–Wallis testing with Bonferroni-adjusted post hoc analyses, adjusted negative binomial regression for length of hospital stay, zero-inflated negative binomial regression for total antibiotic duration, and Firth penalized logistic regression for binary outcomes. **Results:** Overall concordance between intraoperative and histopathological grading was 74.0%. Agreement was moderate by unweighted Cohen’s kappa (0.539) and substantial by weighted kappa (0.643), with intraoperative grading more often overestimating than underestimating histopathological severity. Intraoperative severity showed stronger correlations than histopathological severity with LOS (τ = 0.347 vs. 0.207) and total duration of antibiotic therapy (τ = 0.331 vs. 0.224). Both outcomes showed a non-linear pattern, with patients with a negative appendix having a longer hospital stay and greater antibiotic exposure than those with phlegmonous appendicitis, while advanced disease was associated with the greatest treatment burden. Thirty-day readmission was uncommon and not associated with severity. **Conclusions:** Agreement between intraoperative and histopathological grading was moderate to substantial. Intraoperative grading showed stronger associations with immediate postoperative outcomes than histopathological grading, reflecting its role as the primary driver of clinical decision-making in the immediate postoperative period. These findings do not diminish the diagnostic value of histopathological examination, which remains essential for confirmation and detection of unexpected pathology.

## 1. Introduction

Acute appendicitis is one of the most common reasons for emergency abdominal surgery globally, carrying a lifetime risk of 7–8% and an annual occurrence rate of about 100 cases per 100,000 adults [1]. Diagnosis is based on clinical evaluation, combined with laboratory findings, scoring systems, and imaging modalities, and there is emerging interest in the use of artificial intelligence and machine learning in diagnosis [2]. Despite these advances, accurate diagnosis remains difficult, with potentially missed rates of 6% in adults and 4% in children and increased risk depending on sex, age, constipation, and comorbidities [3].

According to the 2025 WSES Jerusalem Guidelines for acute appendicitis, management should be guided by clinical risk stratification and imaging, followed by treatment tailored to disease severity. In patients with uncomplicated acute appendicitis, both laparoscopic appendectomy and nonoperative management with antibiotics are acceptable options, with shared decision-making recommended when appropriate, while laparoscopic appendectomy remains the preferred surgical approach. This is particularly true in cases of gangrene or perforation, and immediate surgery is advised for a perforated appendicitis. The guidelines also support limited postoperative antibiotic use, recommending single-shot prophylaxis for uncomplicated cases and short-course therapy for complicated appendicitis, while patients presenting with appendiceal abscess may be managed nonoperatively, with interval appendectomy considered in selected cases [4]. Based on a meta-analysis of randomized trials in adults, antibiotic treatment for uncomplicated appendicitis appears to have similar short-term treatment success and major complication rates compared to surgery, but is associated with longer hospital stay and a median cumulative recurrence rate of 18%. In contrast, a large 2025 pediatric randomized trial found antibiotics to be inferior to appendicectomy, with treatment failure at 1 year in 34% of children treated with antibiotics versus 7% of those undergoing surgery [5,6]. In a series of incidental appendectomies, it has been shown that 25 incidental appendectomies have to be performed to detect one neoplasm of the appendix [7]. However, in carefully selected patients, antibiotic-only treatment is also possible without risking missing aggressive appendiceal tumors [8].

Histopathological examination is considered the gold standard for the definitive diagnosis of appendicitis, as it allows characterization of the extent of inflammation, from mucosal involvement to transmural and gangrenous findings [9,10]. Several studies have examined the concordance between intraoperative and histopathological assessment of acute appendicitis and found that agreement is lower in advanced than in uncomplicated disease. In a mixed adult and children multicenter cohort, agreement was 93.5% for intraoperatively uncomplicated appendicitis but only 46.6% for intraoperatively complicated appendicitis, and postoperative outcomes remained more closely related to the intraoperative classification than to the histological classification [11]. Pediatric-focused studies have also reported variable agreement, with operative assessment more closely reflecting clinical outcomes such as infectious complications. Furthermore, underestimation of severity during surgery has been associated with longer hospital stay and a higher complication rate [12,13].

Post-appendectomy management is largely shaped by what the surgeon observes during surgery, since these impressions might determine how long antibiotics are continued and when patients are discharged [13,14]. Yet histopathology reports frequently uncover findings such as inflammation or tumors that might not be apparent during surgery [15].

This poses an important clinical question: if intraoperative assessment drives early postoperative management, the practical value of histopathology for immediate decision-making may be limited, even though it remains essential for diagnostic confirmation and detection of unexpected pathology. Clarifying the relationship between intraoperative and histopathological severity is therefore relevant not only for diagnostic concordance, but also for antibiotic stewardship, discharge planning, and interpretation of postoperative outcomes. Therefore, the aim of this study was to evaluate the agreement between intraoperative and histopathological grading of acute appendicitis and to examine how each relates to postoperative clinical outcomes, particularly length of hospital stay (LOS) and duration of antibiotic therapy. In addition, we explored whether these relationships differed between pediatric and adult patients and whether appendicitis severity was associated with 30-day readmission and postoperative complications.

## 2. Methods

### 2.1. Study Design and Setting

This retrospective study included pediatric and adult patients who underwent appendectomy for suspected acute appendicitis at the Department of Pediatric Surgery and the Department of Surgery of the University Hospital of Split between 1 January 2020 and 31 December 2025.

### 2.2. Study Population

**Figure 1:**
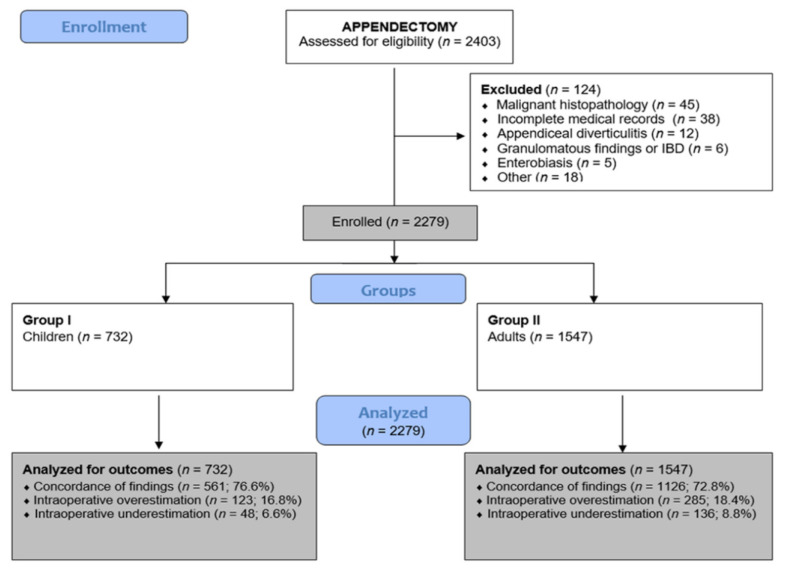
Flowchart of patient selection.

All patients operated on for a clinical diagnosis of acute appendicitis were considered eligible for inclusion. Patients who underwent incidental appendectomy were not included in the study. Furthermore, patients with unavailable medical documentation or missing essential variables required for the analysis, particularly intraoperative or histopathological findings, and patients with other appendiceal or intestinal conditions, including enterobiasis, inflammatory bowel disease, endometriosis, diverticulitis, appendiceal malignancy, chronic appendicitis, or exacerbation of chronic appendiceal disease, were excluded. During the study period, data were identified for 2403 patients. After application of the exclusion criteria, the final study cohort consisted of 2279 patients (Figure 1).

Histopathological findings were classified as negative (absence of acute inflammatory changes of the appendix in the resected specimen), phlegmonous appendicitis (neutrophilic infiltration of the appendiceal wall), or gangrenous appendicitis (necrosis of the appendiceal wall). Intraoperative findings were classified as negative (macroscopically without feature of acute inflammation), phlegmonous, gangrenous, or gangrenous perforated appendicitis. Negative appendectomy cases were retained in the analyses because all patients underwent surgery for suspected acute appendicitis and because this subgroup was clinically relevant to diagnostic concordance and postoperative management. For direct comparison between intraoperative and histopathological grading, the gangrenous and gangrenous perforated intraoperative categories were collapsed into a single severe category, because histopathological reports did not consistently record perforation status and therefore did not provide an equivalent, separate perforated category. This merging was used only for concordance analyses. For analyses of postoperative outcomes based on intraoperative findings, gangrenous and gangrenous perforated appendicitis were retained as separate categories to preserve clinically relevant differences. Intraoperative grading was based on the operating surgeon’s macroscopic assessment and description in the operative report and reflected routine clinical practice during the study period in which appendicitis severity was graded as normal, phlegmonous, gangrenous, or gangrenous perforated. A prospectively standardized intraoperative scoring system was not used, and interobserver variability was not assessed.

### 2.3. Data Collection

Data were obtained from the hospital information system and by review of archived medical records. Demographic and clinical variables included age, sex, age group (pediatric vs. adult), intraoperative findings, histopathological diagnosis, surgical approach, abdominal drain placement, LOS, use of in-hospital antibiotics, duration of antibiotics prescribed at discharge, total duration of antibiotic therapy, 30-day readmission, and 30-day postoperative complications.

Total duration of antibiotic therapy was defined as the sum of in-hospital antibiotic exposure starting from date of hospitalization and the number of days of antibiotics prescribed at discharge. Data on the duration of antibiotic therapy prescribed at discharge were missing for 143 patients (6.3%), whereas all other analyzed variables were either complete or had less than 1.3% missing data.

### 2.4. Study Aims and Outcomes

The primary aim of the study was to evaluate the agreement between intraoperative and histopathological grading of acute appendicitis. The secondary aim was to compare the association of intraoperative and histopathological severity with postoperative clinical outcomes, particularly LOS and duration of antibiotic therapy.

Additional exploratory analyses evaluated whether these associations differed between pediatric and adult patients and whether appendicitis severity was associated with 30-day readmission and 30-day postoperative complications.

### 2.5. Statistical Analysis

Agreement between intraoperative and histopathological classifications was assessed using Cohen’s kappa coefficient and weighted Cohen’s kappa. Concordant and discordant cases were additionally categorized as intraoperative overestimation or underestimation relative to histopathology.

Associations between appendicitis severity and postoperative outcomes were first examined using non-parametric correlation analysis with Kendall’s tau coefficient, which was chosen because appendicitis severity was treated as an ordinal variable with a limited number of categories and frequent tied values. These analyses were performed overall and separately in pediatric and adult subgroups. To compare the strength of correlations between children and adults, bootstrap resampling with 2000 iterations was used to estimate the distribution of the difference in Kendall’s tau coefficients and corresponding 95% confidence intervals.

Differences in continuous outcomes across severity categories were evaluated using the Kruskal–Wallis test. When appropriate, post hoc pairwise comparisons were performed with Bonferroni correction. Because visual inspection and descriptive summaries indicated non-normal and skewed distributions for hospital stay and antibiotic-duration variables, non-parametric methods were used for primary group comparisons.

To further explore non-linear relationships between appendicitis severity and postoperative outcomes, intraoperative and histopathological severity were also modelled as categorical predictors in adjusted regression analyses. Because LOS and total antibiotic duration are overdispersed count outcomes, negative binomial regression was used for LOS, and zero-inflated negative binomial regression was used for total antibiotic duration, with adjustment for sex and age group. Negative appendectomy cases were used as the reference category. Thirty-day readmission and postoperative complications were analyzed as binary outcomes. Associations with severity category were first assessed using chi-square testing or Fisher’s exact test when expected cell counts were small, because 30-day complications and readmission were sparse binary outcomes. Firth penalized logistic regression was used to reduce small-sample bias, fitted separately for intraoperative and histopathological severity, with adjustment for sex and age group. Results were reported as odds ratios with 95% profile likelihood confidence intervals. All regression analyses were performed using complete case data for the variables included in each model. No imputation was performed for antibiotic duration outcomes, and, considering the relatively small proportion of patients with missing data, patients with missing discharge antibiotic duration data were excluded only from analyses requiring total antibiotic duration.

Because drain placement may influence LOS and antibiotic duration, sensitivity analyses were performed by additionally including drain placement in the aforementioned models, as drain placement may act both as a confounder and as a mediator of the relationship between intraoperative severity and postoperative outcomes.

Statistical analysis was performed using R software, version 4.4.0 (R Foundation for Statistical Computing, Vienna, Austria). All tests were two-sided, and *p* < 0.05 was considered statistically significant.

### 2.6. Ethics

This study was conducted in accordance with the principles of the Declaration of Helsinki and applicable national regulations governing the protection of patient rights and personal data. Ethical approval was granted by the Ethics Committee of the University Hospital of Split (approval number 520-03/25-01-100, approved on 31 March 2025).

## 3. Results

Overall demographic and clinical characteristics are presented in Table 1. Among 2279 patients included in the final analysis, 1346 (59.1%) were male, and the median age was 28 years (IQR 15–47). The mean LOS was 3.82 ± 3.65 days, with a median of 3 days (IQR 2–4). An abdominal drain was placed in 352 patients (15.5%), and laparoscopy was the dominant surgical approach (92.3%). Compared with adults, children had a longer hospital stay, far less frequent drain placement, and a similar rate of laparoscopic surgery, with lower conversion rates to open surgery.

**Figure 2:**
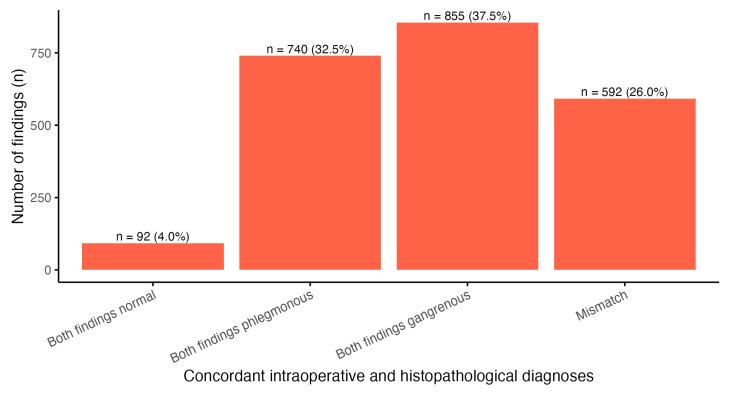
Concordance between intraoperative and histopathological grading of acute appendicitis. Distribution of concordant and discordant classifications between intraoperative and histopathological findings after merging the gangrenous and gangrenous perforated intraoperative categories into a single severe category. Bars represent the number and proportion of cases in each concordance category.

After combining the gangrenous and gangrenous perforated intraoperative categories for comparison with histopathology, overall concordance was observed in 1687 cases (74%). Agreement between intraoperative and histopathological grading was moderate based on unweighted Cohen’s κ (0.539, 95% CI 0.507–0.571) and substantial based on weighted κ (0.643, 95% CI 0.614–0.671); both were statistically significant (*p* < 0.001), with similar agreement in children (κ 0.569, 95% CI 0.514–0.624; weighted κ 0.642, 95% CI 0.593–0.691) and in adults (κ 0.520, 95% CI 0.481–0.559; weighted κ 0.642, 95% CI 0.606–0.677). In discordant cases, intraoperative grading more often overestimated than underestimated histopathological severity (17.9% vs. 8.1%) (Figure 2, Table 2).

**Figure 3:**
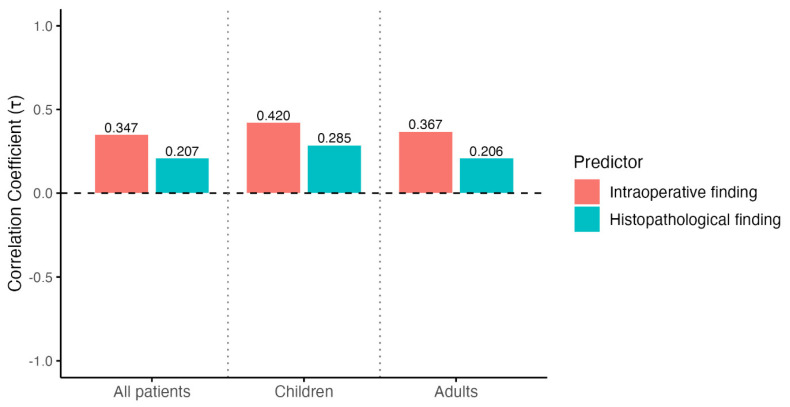
Correlation between appendicitis severity and length of hospital stay (LOS). Kendall’s tau correlation coefficients are shown for the association between appendicitis severity and LOS in all patients, children, and adults. Red bars represent intraoperative findings, and teal bars represent histopathological findings.

Disease severity correlated positively with LOS, and this association was consistently stronger for intraoperative grading than for histopathological grading. In the overall cohort, Kendall’s tau was 0.347 for intraoperative severity and 0.207 for histopathological severity. The same pattern was observed in children (τ = 0.420 vs. 0.285) and in adults (τ = 0.367 vs. 0.206) (Figure 3).

The mean number of prescribed post-discharge antibiotic days was 3.25 ± 3.01, with a median of 5 days (IQR 0–5). The mean total duration of antibiotic therapy was 5.95 ± 4.8 days, with a median of 7 days (IQR 0–9). Adults had longer prescribed antibiotic treatment and greater total antibiotic exposure than children (Table 3).

**Figure 4:**
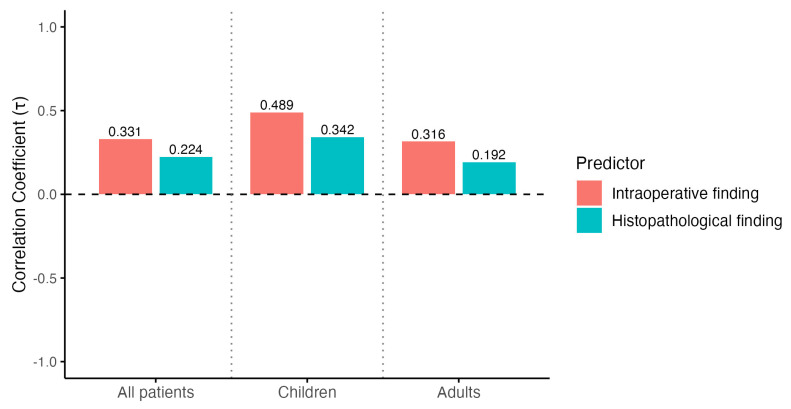
Correlation between appendicitis severity and total duration of antibiotic therapy. Kendall’s tau correlation coefficients for the association between appendicitis severity and total duration of antibiotic therapy in all patients, children, and adults. Red bars represent intraoperative findings, and teal bars represent histopathological findings.

As with hospital stay, the correlation between disease severity and total duration of antibiotic therapy was stronger for intraoperative grading than for histopathological grading overall (τ = 0.331 vs. 0.224), in children (τ = 0.489 vs. 0.342), and in adults (τ = 0.316 vs. 0.192) (Figure 4).

**Figure 5:**
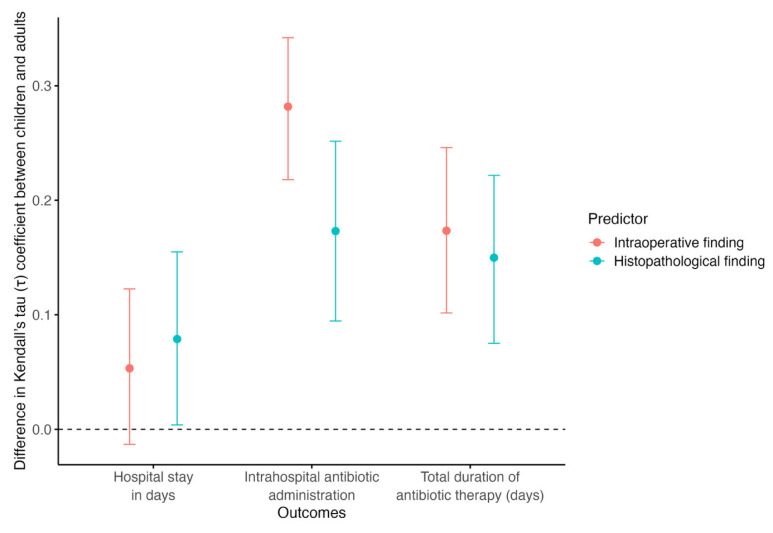
Bootstrap estimated differences in Kendall’s tau coefficients between pediatric and adult patients for postoperative outcomes. Red points with 95% confidence intervals represent intraoperative findings, and teal points with 95% confidence intervals represent histopathological findings. Positive values indicate stronger correlations in children than in adults.

Bootstrap comparison showed little age-group difference in the association between appendicitis severity and LOS. The pediatric–adult difference was not significant for intraoperative grading (Δτ = 0.053, 95% CI −0.013 to 0.123, *p* = 0.127) but was significant for histopathological grading (Δτ = 0.079, 95% CI 0.003 to 0.155, *p* = 0.041). In contrast, age-group differences were significant (*p* < 0.001) for antibiotic-related outcomes, with stronger severity–antibiotic associations in children than adults for both in-hospital antibiotic use and total antibiotic duration, particularly when severity was assessed intraoperatively (Figure 5).

**Figure 6:**
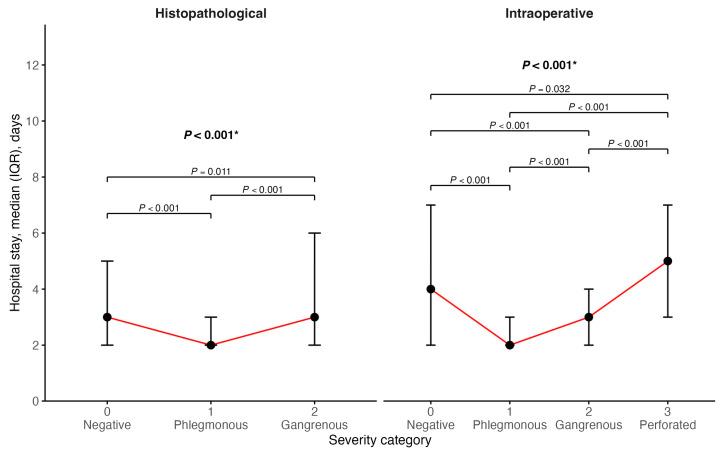
Distribution of length of hospital stay across severity categories according to intraoperative and histopathological grading. IQR—interquartile range. * Kruskal–Wallis test with post hoc Bonferroni test.

LOS differed significantly across severity categories for both intraoperative and histopathological classifications (Kruskal–Wallis *p* < 0.001 for both intraoperative findings and histopathology) (Figure 6). Patients with a normal appendix had longer hospital stays than those with phlegmonous appendicitis. In the intraoperative classification, median hospital stay was 4.0 days (IQR 2.0–7.0) for a normal appendix and 2.0 days (IQR 2.0–3.0) for phlegmonous appendicitis; corresponding mean values were 5.59 ± 4.95 and 2.72 ± 2.39 days. Patients with more advanced disease again showed prolonged hospitalization, with a median stay of 3.0 days (IQR 2.0–4.0) for gangrenous appendicitis and 5.0 days (IQR 3.0–7.0) for gangrenous perforated appendicitis. A similar but less pronounced pattern was seen in histopathological grading, where the median hospital stay was 3.0 days (IQR 2.0–5.0) for a normal appendix, 2.0 days (IQR 2.0–3.0) for phlegmonous appendicitis, and 3.0 days (IQR 2.0–6.0) for gangrenous appendicitis. Bonferroni-adjusted pairwise comparisons showed a marked difference between the normal appendix group and the appendicitis groups (*p* < 0.05) (Figure 6).

To account for this non-linearity, severity was additionally modelled as a categorical variable in regression analyses, with negative appendectomy as a baseline. Negative binomial regression confirmed the non-linear pattern for LOS. Compared with patients with a normal appendix, those with phlegmonous appendicitis had significantly shorter hospital stays in both the intraoperative model (incidence rate ratio (IRR) 0.457, 95% CI 0.408–0.512, *p* < 0.001) and the histopathological model (IRR 0.703, 95% CI 0.638–0.776, *p* < 0.001). In the intraoperative model, gangrenous appendicitis was also associated with shorter hospitalization than a normal appendix (IRR 0.602, 95% CI 0.536–0.676, *p* < 0.001), whereas perforated appendicitis did not differ significantly (IRR 1.038, 95% CI 0.926–1.164, *p* = 0.521). In the histopathological model, gangrenous appendicitis was not significantly different from the normal appendix group (IRR 1.041, 95% CI 0.946–1.146, *p* = 0.411). Adults had shorter stays than children in both models (intraoperative: IRR 0.746, 95% CI 0.706–0.787, *p* < 0.001; histopathological: IRR 0.780, 95% CI 0.736–0.827, *p* < 0.001). Sex was not significantly associated with LOS in either model (*p* = 0.409 and *p* = 0.268, respectively). Zero-inflated negative binomial regression for total antibiotic duration showed that, in the count component, phlegmonous appendicitis was associated with fewer antibiotic days than a normal appendix in the intraoperative model (IRR 0.692, 95% CI 0.633–0.757, *p* < 0.001), as was gangrenous appendicitis (IRR 0.816, 95% CI 0.746–0.893, *p* < 0.001). Perforated appendicitis did not significantly differ from the normal appendix group in the count component (IRR 1.078, *p* = 0.099). Adults received longer antibiotic courses than children (intraoperative count model: IRR 1.264, 95% CI 1.204–1.327, *p* < 0.001). In the zero-inflation component, gangrenous (OR 0.419, 95% CI 0.256–0.683, *p* < 0.001) and perforated appendicitis (OR 0.175, 95% CI 0.102–0.302, *p* < 0.001) were strongly associated with reduced probability of receiving zero antibiotic days, while adults were far less likely to receive no antibiotics than children (OR 0.178, 95% CI 0.144–0.220, *p* < 0.001). In the histopathological model, phlegmonous appendicitis was similarly associated with fewer antibiotic days than a normal appendix in the count component (IRR 0.829, 95% CI 0.767–0.896, *p* < 0.001), whereas gangrenous appendicitis did not differ significantly (IRR 1.045, 95% CI 0.969–1.126, *p* = 0.250). In the zero-inflation component, gangrenous histopathology was associated with a substantially reduced probability of receiving zero antibiotic days (OR 0.279, 95% CI 0.196–0.396, *p* < 0.001), phlegmonous appendicitis showed a smaller but significant effect (OR 0.712, 95% CI 0.510–0.994, *p* = 0.046), and adults were far less likely to receive no antibiotics than were children (OR 0.181, 95% CI 0.147–0.222, *p* < 0.001). Sex was not significantly associated with total antibiotic duration in either zero-inflated negative binomial regression model.

**Figure 7:**
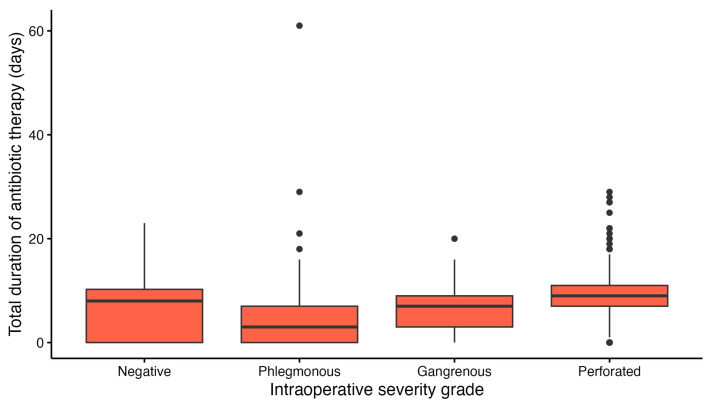
Distribution of total duration of antibiotic therapy in days across intraoperative severity categories.

A similar non-linear pattern was observed for total antibiotic exposure (Figure 7). The total duration of antibiotic therapy differed significantly across intraoperative severity categories (Kruskal–Wallis *p* < 0.001). Patients with phlegmonous appendicitis had the shortest treatment duration (mean 3.98 ± 4.40 days; median 3 days, IQR 0–7), whereas patients with perforated appendicitis had the longest antibiotic exposure (mean 9.25 ± 4.47 days; median 9 days, IQR 7–11). Patients with a macroscopically normal appendix also had relatively prolonged antibiotic treatment (mean 6.82 ± 5.38 days; median 8 days, IQR 0–10.25), exceeding that observed in the phlegmonous group and approaching the gangrenous category (mean 6.28 ± 3.90 days; median 7 days, IQR 3–9).

Thirty-day readmission was uncommon and did not differ significantly according to either intraoperative or histopathological severity grading. Readmission rates ranged from 1.2% to 2.3% across intraoperative groups and from 1.1% to 2.4% across histopathological groups. Fisher’s exact testing showed an insignificant association between the severity category and readmission (intraoperative *p* = 0.331; histopathology *p* = 0.141). In the adjusted Firth penalized logistic regression, neither intraoperative severity (phlegmonous: OR 0.470, 95% CI 0.137–2.447, *p* = 0.324; gangrenous: OR 0.618, 95% CI 0.174–3.256, *p* = 0.521; perforated: OR 0.961, 95% CI 0.281–4.987, *p* = 0.955) nor histopathological severity (phlegmonous: OR 0.363, 95% CI 0.134–1.114, *p* = 0.074; gangrenous: OR 0.697, 95% CI 0.285–2.013, *p* = 0.475) was independently associated with 30-day readmission. Sex and age group were also not significantly associated with readmission in either model.

Thirty-day postoperative complications differed significantly across severity categories in the unadjusted analyses. According to intraoperative grading, complication rates were 8.3% in patients with a negative appendix, 4.1% in phlegmonous appendicitis, 4.8% in gangrenous appendicitis, and 7.0% in perforated appendicitis. According to histopathological grading, complication rates were 7.6% in negative cases, 4.0% in phlegmonous cases, and 5.9% in gangrenous cases. These differences were statistically significant in categorical analyses (intraoperative: χ^2^ *p* = 0.043; histopathology: χ^2^ *p* = 0.042). In adjusted Firth penalized logistic regression, histopathological phlegmonous appendicitis was the only severity category independently associated with 30-day complications: patients with phlegmonous histopathology had significantly lower odds of complications compared with those with a histologically normal appendix (OR 0.507, 95% CI 0.285–0.941, *p* = 0.032). No other severity category reached significance in the histopathological model (gangrenous: OR 0.736, 95% CI 0.427–1.337, *p* = 0.302), nor in the intraoperative model (phlegmonous: OR 0.470, 95% CI 0.234–1.040, *p* = 0.061; gangrenous: OR 0.547, 95% CI 0.265–1.232, *p* = 0.139; perforated: OR 0.806, 95% CI 0.396–1.797, *p* = 0.579). Sex and age group were not significantly associated with complications in either model. The direction of the phlegmonous association, where fewer complications were noted than the normal appendix group, mirrors the non-linear pattern observed for length of stay.

Sensitivity analyses additionally adjusting for drain placement are summarized in Table 4. Inclusion of drain placement attenuated some severity effect estimates but did not change the overall findings in regards to severity category. Drain placement was independently associated with longer LOS and longer total antibiotic duration, but it was not independently associated with 30-day readmission or postoperative complications.

## 4. Discussion

In this large, single-center cohort, intraoperative and histopathological grading of acute appendicitis showed only moderate-to-substantial agreement, indicating that the two assessments capture overlapping but not identical aspects of disease severity. Intraoperative grading was more closely associated with early postoperative outcomes, particularly length of stay and antibiotic exposure, which is clinically plausible because operative findings directly inform immediate postoperative decision-making. Accordingly, operative and histopathological assessments should not be regarded as interchangeable: histopathology remains essential for definitive diagnosis, whereas intraoperative assessment appears to have greater relevance for early postoperative management. This interpretation is consistent with previous reports suggesting that surgical assessment correlates more closely than histopathology with short-term treatment decisions and postoperative course [16].

A systematic review and meta-analysis showed that surgeons have high intraoperative sensitivity for distinguishing an abnormal from a normal appendix, whereas specificity is lower. However, these findings relate to a binary classification and do not address discrimination among appendicitis classifications. Moreover, surgeons were slightly more sensitive and specific in a pediatric population [17]. Similarly, studies have shown that overall concordance among surgeons significantly differs, indicating different knowledge levels among surgeons, but operator experience does not affect the accuracy of the intraoperative assessment [18,19,20]. Bolmers et al. demonstrated that discrepancies between intraoperative and histological classification are common, especially in patients judged intraoperatively to have complicated appendicitis, and showed that postoperative outcomes aligned more closely with the intraoperative impression than with histology alone [11]. Similarly, pediatric studies have reported only moderate concordance between intraoperative and histopathological classifications. Intraoperative assessment appears to correlate more closely than histopathology with clinically relevant postoperative outcomes, including length of hospital stay, while underestimation of disease severity during surgery has been associated with longer hospitalization. Agreement has also been shown to be weaker for the overall diagnosis of appendicitis than for classification as perforated or non-perforated disease, with no clear influence of surgeon experience or operative approach [13,21,22]. Our findings extend these observations by showing a similar pattern in a large combined pediatric and adult cohort and by demonstrating that this difference is particularly evident for management-related outcomes such as antibiotic exposure and hospital stay.

One likely explanation for the tendency of intraoperative grading to exceed histopathological severity is that the surgeon evaluates the entire operative field rather than the appendix specimen alone. Presence of purulent fluid, fibrin deposits, periappendiceal abscess, localized or diffuse peritonitis, and tissue friability might influence future decision and postoperative course. Furthermore, differences in surgeon experience, interpretation of periappendiceal contamination, and documentation style can all contribute to discordance with histopathology. Histopathology, by contrast, is limited to microscopic assessment of the excised appendix and cannot directly account for the extent of peritoneal contamination. This difference in perspective may explain why operative assessment more frequently overestimates disease severity, while still remaining more closely linked to short-term postoperative management. Some degree of discrepancy may also reflect the absence of fully standardized intraoperative grading criteria and unavoidable inter-surgeon variability, which was not analyzed.

A particularly relevant finding from the present study is that the relationship between appendiceal severity and postoperative burden was not linear. Patients with phlegmonous appendicitis had the shortest LOS and lowest antibiotic exposure, whereas patients with advanced disease had the greatest treatment burden. More unexpectedly, patients with a negative appendix also showed relatively prolonged hospitalization and antibiotic use. This is in contrast with some studies that found shorter LOS in a negative appendectomy group [23]. However, other series have also reported longer hospital stays and higher morbidity with negative appendectomy than in patients with confirmed uncomplicated appendicitis [24,25]. These contradictory findings could reflect differences in institutional negative appendectomy rates, patient case mix, and the underlying complexity of cases in which the decision to operate was made despite a subsequently normal appendix. In such patients, postoperative recovery may be prolonged not because of appendiceal inflammation itself, but because symptoms, fever, abdominal pain, or inflammatory markers persist and prompt further observation, additional imaging, or consultation. In at least some cases, alternative diagnoses such as mesenteric lymphadenitis, enterocolitis or ileocolitis, urinary tract infection, gynecologic pathology, or other inflammatory intra-abdominal conditions may explain the prolonged hospital stay and continued antibiotic use [25]. Thus, the unexpectedly long LOS and antibiotic exposure in the negative appendectomy group do not imply that a negative appendectomy represents more severe appendiceal condition, but likely reflect diagnostic complexity and uncertainty rather than disease severity in the conventional sense. This interpretation also helps explain why the phlegmonous group may have had the most predictable postoperative course: once the diagnosis is clear and the disease is uncomplicated, clinical recovery and discharge planning may follow a more standardized pathway. Nevertheless, these findings should be interpreted cautiously, as they may represent a specific feature of our cohort depending on the local postoperative management pathways, as some studies have shown it could be possible to reduce LOS in children with uncomplicated appendicitis through the use of specific clinical pathways [26,27].

The stronger association of intraoperative severity with LOS and antibiotic exposure likely reflects not only disease appearance, but also the fact that operative findings are the information available at the time early postoperative decisions are made. In this sense, intraoperative grading may be a better predictor of short-term management–related outcomes, whereas histopathology retains its central role in confirming the diagnosis and identifying unexpected pathology.

The antibiotic-related findings are particularly relevant in the current era of antimicrobial stewardship. Recent studies increasingly support shorter postoperative antibiotic strategies in selected patients with complicated appendicitis [28,29,30]. Furthermore, postoperative antibiotics do not improve outcomes in children with nonperforated appendicitis [31,32]. Although our study does not evaluate specific postoperative antibiotic protocols, it reflects real-world practice in which antibiotic duration and discharge timing were determined by the leading surgeon. Therefore, the association between intraoperative severity and antibiotic exposure should not be interpreted only as a biological relationship between disease severity and treatment need. It also likely reflects the fact that surgeons use intraoperative findings, together with postoperative clinical recovery, to decide whether antibiotics should be continued and when patients could be discharged. Practically, this means that decisions regarding continuation or discontinuation of postoperative antibiotics are likely to be made before pathology becomes available and should therefore be guided by operative severity and the patient’s clinical course. Histopathology remains important for diagnostic confirmation, but it appears to play a limited role in immediate antibiotic decision-making. Therefore, the practical role of histopathology is different from that of intraoperative grading. It is unlikely to alter early postoperative treatment in most patients, because results are usually received after discharge, but it remains clinically important for confirming the diagnosis or identifying unexpected findings. These data therefore support the development of clearer postoperative pathways in which standardized intraoperative classification is explicitly linked to antibiotic duration and discharge criteria.

The age-related findings also deserve comment. Although concordance between intraoperative and histopathological grading was only slightly stronger in children than in adults, bootstrap analysis showed that the association between severity and antibiotic-related outcomes was stronger in children, particularly when severity was assessed intraoperatively. This suggests that, although the general relationship between operative and pathological grading is similar in both age groups, the clinical consequences of severity classification may differ between pediatric and adult practice. In our cohort, adults had shorter hospital stays but longer total antibiotic treatment durations than children, likely reflecting differences in postoperative pathways, discharge thresholds, and prescribing practices between the two populations. Also, that implies that antibiotic prescribing pattern in children means that the decision to prescribe at all is tightly linked to disease severity as perceived intraoperatively. Drain placement was more frequent in adults and is particularly difficult to interpret in a retrospective study, because it may function both as a marker of more severe intraoperative disease and as an intervention that can independently influence postoperative recovery, discharge timing, and antibiotic duration. Therefore, part of the association between intraoperative severity and postoperative outcomes may be mediated or confounded by drain use. To explore this, sensitivity analyses were performed, with drain placement added as an additional covariate to the regression models. The main conclusions were unchanged, although some severity-effect estimates were attenuated. Drain placement was independently associated with longer LOS and longer total antibiotic duration, but it was not significantly associated with 30-day readmission or postoperative complications. These findings suggest that part of the observed increase in LOS and antibiotic exposure in more severe cases may reflect management decisions, particularly drain placement, rather than underlying disease severity alone. Recent evidence does not support a clear clinical benefit of routine drainage after appendectomy for complicated appendicitis and suggests that drainage may prolong recovery and length of stay, although the certainty of this evidence remains very low [33,34]. However, one study found that prophylactic drain placement with irrigation may reduce abscesses in severe uncontained perforated appendicitis [35]. Therefore, drain placement should be considered when interpreting the association among appendicitis severity, LOS, and antibiotic exposure, as it may independently prolong recovery while also acting as a marker of more complex disease or surgical decision making.

The association between appendicitis severity and 30-day outcomes was more limited. Readmission was uncommon and was not associated with either intraoperative or histopathological severity. Postoperative complications showed some unadjusted differences across categories, but these were largely attenuated after adjustment in the regression analysis. The lower adjusted odds of complications in the histopathological phlegmonous group relative to the normal appendix group should be interpreted cautiously, particularly given the small number of events and the likely heterogeneity of the negative appendectomy subgroup. Overall, these findings suggest that severity classification is more strongly related to immediate postoperative management than to later adverse outcomes.

This study has several strengths, including the large cohort, inclusion of both pediatric and adult patients within the same institutional setting, and analysis of outcomes directly relevant to routine surgical decision-making. However, several limitations should be acknowledged. First, the retrospective design introduces the possibility of selection bias and depends on the completeness and quality of operative and medical documentation. We also could not evaluate the potential influence of operative timing, including day versus night surgery and delay from admission to surgery. Second, intraoperative grading was based on routine surgeon assessment and was not prospectively standardized. Formal interobserver variability between surgeons or between pathologists could not be quantified, and this may have influenced both the observed concordance of findings and the association between intraoperative severity and management-related outcomes. Third, direct comparison between intraoperative and histopathological classifications required collapsing the gangrenous and perforated operative categories into a single severe category, because histopathology did not provide a corresponding perforation variable. This may have reduced granularity and may have obscured clinically important differences within complicated appendicitis. Fourth, postoperative management was not protocolized. Antibiotic prescribing, discharge timing, and drain placement may therefore reflect clinician preference, departmental practice, patient age group, and perceived disease severity. Fifth, the negative appendectomy subgroup was relatively small and likely heterogeneous, which may limit the stability and interpretation of the observed non-linear patterns. Finally, length of stay and antibiotic exposure are not purely biological outcomes, but are also shaped by the same intraoperative impressions that clinicians use to guide management. This may partly explain why intraoperative grading showed stronger associations with these endpoints than histopathology. In addition, the low number of readmissions and complications limits the precision of inference for 30-day outcomes.

Overall, the present study supports a complementary rather than competing role for intraoperative assessment and histopathology. Histopathology remains essential for diagnostic confirmation and detection of unexpected pathology, whereas intraoperative assessment appears to be more relevant for early postoperative decision-making, particularly with respect to antibiotic use and discharge planning. Future prospective studies should include factors such as time of surgery and interobserver variability and evaluate standardized intraoperative grading systems linked to predefined postoperative care pathways in order to improve treatment consistency, reduce unnecessary antibiotic exposure, and shorten hospitalization without compromising safety.

## 5. Conclusions

In this large, single-center cohort, agreement between intraoperative and histopathological grading of acute appendicitis was moderate to substantial, with intraoperative assessment more often overestimating than underestimating histopathological severity. Intraoperative grading showed stronger associations than histopathological classification with length of hospital stay and total antibiotic exposure, indicating that it is more closely aligned with early postoperative management. Both outcomes also demonstrated a non-linear pattern, as patients with a negative appendix had greater length of hospital stay and antibiotic use than those with phlegmonous appendicitis, likely reflecting diagnostic complexity rather than appendiceal severity alone. Histopathology remains essential for confirming the diagnosis and detecting unexpected pathology, but intraoperative assessment appears to have greater immediate relevance for decisions regarding postoperative antibiotics and discharge. These findings support the development of standardized intraoperative grading systems linked to postoperative care pathways in order to improve treatment consistency, strengthen antibiotic stewardship, and reduce unnecessary hospitalization.

## Figures and Tables

**Table 1:** Demographic and clinical characteristics of patients overall and according to age group.

Figure	All Patients (*n =* 2279)	Distribution by Age Group		
		Children (*n* = 732)	Adults (*n* = 1547)	*p*
Sex, *n* (%)				
Male	1346 (59.1)	471 (64.3)	876 (56.6)	<0.001 *
Female	932 (40.9)	261 (35.7)	671 (43.4)	
Age, years [median, IQR]	28 (15–47)	12 (9–15)	39 (28–56)	<0.001 ^†^
LOS, days [mean (SD)]	3.82 (3.65)	4.39 (2.82)	3.54 (3.95)	<0.001 ^†^
LOS, days [median (IQR)]	3 (2–4)	3 (2–6)	3 (2–4)	<0.001 ^†^
Intraoperative findings, *n* (%)				
Normal	108 (4.7)	21 (2.9)	87 (5.6)	0.004 *
Phlegmonous	1017 (44.7)	383 (52.3)	634 (41)	
Gangrenous	641 (28.1)	194 (26.5)	447 (28.9)	
Gangrenous perforated	513 (22.5)	134 (18.3)	379 (24.5)	
Histopathology findings, *n* (%)				
Normal	210 (9.2)	54 (7.3)	156 (10.1)	0.013 *
Phlegmonous	1044 (45.8)	392 (53.6)	652 (42.1)	
Gangrenous	1025 (45)	286 (39.1)	739 (47.8)	
Drain placed, *n* (%) *				
Yes	352 (15.5)	17 (2.3)	335 (21.8)	0.001 *
No	1918 (84.5)	714 (97.7)	1204 (78.2)	
Surgical approach, *n* (%)				
Open	110 (4.8)	52 (7.1)	58 (3.8)	0.001 ^‡^
Laparoscopic	2096 (92.3)	674 (92.4)	1422 (92.3)	
Conversion to open from laparoscopic	65 (2.9)	4 (0.5)	61 (3.9)	
Complications, *n* (%)	126 (5.5)	34 (4.6)	92 (5.9)	0.204 *
Readmission rate in 30 days, *n* (%)	32 (1.4)	12 (1.6)	20 (1.3)	0.512 *

Data are presented as *n* (%), mean (SD), or median (IQR), as appropriate. Percentages were calculated based on the available data, not the full dataset. In the pediatric population, seven drains were intraabdominally placed, while the others were subcutaneously placed; in the adult population, all drain placements were intraabdominally placed. * Chi square test, ^†^ Wilcoxon test or Student’s *t* test as appropriate, ^‡^ Fisher’s exact test. LOS—length of hospital stay; IQR—interquartile range; SD—standard deviation.

**Table 2:** Concordance, intraoperative overestimation, and intraoperative underestimation in comparison with histopathological grading, overall and according to age group.

	All Patients (*n* = 2279)	Distribution by Age Group		
		Children (*n* = 732)	Adults (*n* = 1547)	*p*
Concordance of findings	1687 (74)	561 (76.6)	1126 (72.8)	0.091 *
Intraoperative overestimation	408 (17.9)	123 (16.8)	285 (18.4)	
Intraoperative underestimation	184 (8.1)	48 (6.6)	136 (8.8)	

Data are presented as *n* (%). Concordance was defined after merging the gangrenous and gangrenous perforated intraoperative categories into a single severe category corresponding to gangrenous histopathological appendicitis. * Chi-square test.

**Table 3:** Antibiotic use overall and according to age group.

Feature	All Patients (*n* = 2279)	Distribution by Age Group		
		Children (*n* = 732)	Adults (*n* = 1547)	*p*
Number of days of prescribed antibiotics [mean (SD)]	3.25 (3.01)	0.53 (1.78)	4.63 (2.53)	<0.001 *
Number of days of prescribed antibiotics [median (IQR)]	5 (0–5)	0 (0)	5 (4–7)	<0.001 *
Total length of antibiotic treatment duration [mean (SD)]	5.95 (4.8)	3.45 (4.57)	7.13 (4.43)	<0.001 *
Total length of antibiotic treatment duration [median (IQR)]	7 (0–9)	0 (0–6)	8 (6–10)	<0.001 *
Intrahospital antibiotics usage, *n* (%) Yes No	1803 (79.6) 462 (20.4)	374 (51.4) 353 (48.6)	1429 (92.9) 109 (7.1)	<0.001 **
Antibiotics at discharge, *n* (%) Yes No	1342 (59.5) 914 (40.5)	73 (10.1) 652 (89.9)	1269 (82.9) 262 (17.1)	<0.001 **
The most common prescribed antibiotics at discharge				
Amoxicillin/clavulanic acid monotherapy, *n* (%)	147 (10.9)	51 (69.9)	96 (7.6)	<0.001 **
Ciprofloxacin monotherapy, *n* (%)	23 (1.7)	2 (2.7)	21 (1.7)	0.359 ***
Dual amoxicillin/clavulanic acid and metronidazole, *n* (%)	72 (5.4)	10 (13.7)	62 (4.9)	<0.001 **
Dual ciprofloxacin and metronidazole, *n* (%)	1051 (78.1)	1 (1.4)	1050 (82.7)	<0.001 ***
Dual ciprofloxacin and amoxicillin/clavulanic acid, *n* (%)	4 (0.2)	0 (0)	4 (0.3)	1 ***

Data are presented as number (%), mean (SD), or median (IQR), as appropriate. * Wilcoxon or Student’s *t*-test as appropriate, ** Chi-square test, *** Fisher’s exact test. IQR—interquartile range; SD—standard deviation.

**Table 4:** Sensitivity analyses additionally adjusted for drain placement.

Outcome	Model and Severity Category	Severity Findings After Adding Drain	Drain Effect	Interpretation
Length of stay	NBR Intraoperative severity	Phlegmonous: IRR 0.508, *p* < 0.001; gangrenous: IRR 0.658, *p* < 0.001; perforated: not significant	IRR 1.419, *p* < 0.001	Drain placement was independently associated with longer LOS
Length of stay	NBR Histopathological severity	Phlegmonous: IRR 0.768, *p* < 0.001; gangrenous: not significant	IRR 1.776, *p* < 0.001	Drain placement was independently associated with longer LOS
Total antibiotic duration	ZINB, count component Intraoperative severity	Phlegmonous: IRR 0.732, *p* < 0.001; gangrenous: IRR 0.852, *p* < 0.001; perforated: not significant	IRR 1.198, *p* < 0.001	Drain placement was independently associated with longer antibiotic duration
Total antibiotic duration	ZINB, count component Histopathological severity	Phlegmonous: IRR 0.868, *p* < 0.001; gangrenous: not significant	IRR 1.330, *p* < 0.001	Drain placement was independently associated with longer antibiotic duration
Total antibiotic duration	ZINB, zero-inflation component; Intraoperative severity	Gangrenous: OR 0.420, *p* < 0.001; perforated: OR 0.174, *p* < 0.001; phlegmonous: not significant	OR 1.134, *p* = 0.526	Drain placement was not independently associated with antibiotic duration
Total antibiotic duration	ZINB, zero-inflation component; Histopathological severity	Phlegmonous: OR 0.710, *p* = 0.047; gangrenous: OR 0.283, *p* < 0.001	OR 0.702, *p* = 0.056	Drain placement was not independently associated with antibiotic duration
30-day readmission	FPLR Intraoperative severity	No severity category was significant	OR 2.014, *p* = 0.124	Drain placement was not independently associated with readmission
30-day readmission	FPLR Histopathological severity	No severity category was significant	OR 2.107, *p* = 0.077	Drain placement was not independently associated with 30-day readmission
30-day postoperative complications	FPLR Intraoperative severity	No severity category was significant	OR 1.389, *p* = 0.208	Drain placement was not independently associated with complications
30-day postoperative complications	FPLR Histopathological severity	Phlegmonous: OR 0.532, *p* = 0.047; gangrenous: not significant	OR 1.554, *p* = 0.071	Drain placement was not independently associated with complications.

FPLR—Firth penalized logistic regression, IRR—incidence rate ratio, LOS—length of hospital stay, NBR—negative binominal regression, OR—odds ratio, ZINB—zero-inflated negative binominal regression.

## Data Availability

The data supporting the findings from this study are available from the corresponding author upon reasonable request.
